# The role of patient volunteers in Fangcang Shelter Hospital during the Omicron wave of COVID-19 pandemic in Shanghai, China: a qualitative study

**DOI:** 10.3389/fpubh.2023.1215030

**Published:** 2023-10-12

**Authors:** Lingyan Zhu, Mengqi Xu, Tiantian Ruan, Xiaoli Huang, Jue Cen

**Affiliations:** ^1^Department of Nursing, Shanghai Sixth People's Hospital Affiliated to Shanghai Jiaotong University School of Medicine, Shanghai, China; ^2^School of Nursing, Shanghai Jiaotong University, Shanghai, China; ^3^Department of Cardiology, Shanghai Sixth People's Hospital Affiliated to Shanghai Jiaotong University School of Medicine, Shanghai, China; ^4^Office of Administration, Shanghai Sixth People's Hospital Affiliated to Shanghai Jiaotong University School of Medicine, Shanghai, China; ^5^China Hospital Development Institute, Shanghai Jiaotong University, Shanghai, China

**Keywords:** COVID-19, patient volunteers, qualitative study, omicron, China

## Abstract

**Objective:**

During the Omicron wave of the COVID-19 pandemic in Shanghai, Fangcang Shelter Hospital (FSH) served as the major way in patient quarantine. Many COVID patients served as volunteers in FSH providing a lot of assistance for the medical workers and other COVID patients. The aim of this study was to explore the experiences of patient volunteers in FSH. It helps health professionals better understand their motivational incentives and barriers in their volunteer work, and improves recruiting and managing volunteers in subsequent public health emergencies.

**Methods:**

This is a qualitative study using semi-structured interviews. Thirteen patient volunteers working in an FSH in Shanghai were included. Thematic analysis was applied to data analysis.

**Results:**

Four themes and nine subthemes were identified. The wishes to give back to society and the responsibility of politics and religion were the main reasons for the patients to serve as volunteers in FSH. The patient volunteers served as the bridge to reduce the communication barriers between other patients and healthcare professionals. They also provided support in supply distribution and psychological counseling. They viewed voluntary work as a usual task and tried to solve the barriers in their work. In turn, the voluntary work brought them benefits in mental and physical health, as well as another chance for growth.

**Conclusion:**

Working as volunteers in FSHs not only brought personal benefits to the COVID patients but also fulfilled the needs of the healthcare system during public health emergencies. The mode of mutual help between patients could be taken as an example in other public health emergencies.

## 1. Background

COVID-19 was caused by SARS-CoV-2 and initially emerged in late December 2019 in Wuhan, China. The WHO declared the pandemic as a Public Health Emergency of International Concern in January 2020 (1). During 2022, the SARS-CoV-2 Omicron variant has widely spread with over 600,000,000 infection cases and over 6,000,000 deaths around the world. In Shanghai, there was an Omicron outbreak from March 2022 to June 2022 infecting over 600,000 people in this city (2).Various measures were taken to try to control the Omicron outbreak in Shanghai.

The establishment of Fangcang Shelter Hospital (FSH) for asymptomatic or mildly symptomatic patients was one of the most common pandemic prevention and control measures. During the pandemic in Shanghai, a total of more than 100 FSHs were established in different districts. The FSH at the study site ran from 9 April to 7 May. All 1,356 beds in three wards received a total of 1,351 COVID-19 patients. There were 207 medical staff and 29 logistical staff working at the FSH. The logistics staff included security guards, cleaners, and sterilizers. 21 patients worked as patient volunteers. They first signed an informed consent form. The patient volunteers were then trained in two methods. One is face-to-face at the FSH by the headquarters. The other is online by Shanghai municipal health officials who were in charge of this FSH. After the training, they had to pass a practical examination by the trainers. A training manager supervised their work when they started volunteering. They worked four shifts a day. Although the volunteers did not have insurance, they were provided with protective equipment, such as protective clothing, masks, disinfectants, etc. In addition, the health workers paid close attention to changes in their physical condition and stopped volunteering immediately if they felt unwell. Patient volunteers were allowed to stop volunteering at any time. During the pandemic, the whole community was in a special state. The FSHs urgently needed a large number of logistical staff. The first priority was to ensure that staff were available for food supply, waste collection, disinfection, etc.

In China, FSH served as a major method to keep COVID patients apart from their families and community (3–5). More than 100 FSHs were built during the Shanghai pandemic providing almost 300,000 beds in total (6). During this period, over 40,000 medical staff from 15 provinces of China worked together to support the treatment of COVID-19 patients in Shanghai (7). They were not only at high risk of infection but also bearing great workload and pressure (8, 9). There came a point at which patients’ needs outpaced the capacity of medical staff and managers in FSHs, so non-professionals had to be recruited to make up for the shortage of the healthcare workforce. In addition to the non-COVID volunteers, COVID patients without serious conditions were encouraged to participate in voluntary work and worked as a complementary workforce to medical staff and other workers in FSHs giving assistance to other COVID patients. Patient volunteers completing registration would participate in a 1-h, virtual, or offline training session on contact tracing that covered China Centers for Disease Control and Prevention (CDC) guidelines and privacy protection regulations. After training and examinations, they took over the voluntary work in FSHs. Attention has been paid to the experience and psychological well-being of COVID patients, medical workers, and the general population (10–12).

Existing studies of healthcare workers and student volunteers have shown that age, psychological stress, previous volunteer experience, perceived knowledge of disease prevention, safety concerns, and social support are associated with willingness to serve on the front lines of public health emergencies (13–15). However, as a new and special group that emerged in the prevention and control of the COVID-19 pandemic in China, patient volunteers did not receive enough attention.

In this study, patient volunteers working at the FSH during the pandemic in Shanghai are interviewed. Their perspectives and experiences were characterized to understand the facilitators and barriers of their work. The study helps health professionals better understand their experiences, address their needs, and improve recruiting and managing volunteers in subsequent public health emergencies.

## 2. Methods

We conducted the explorative, qualitative research according to Consolidates Criteria for Reporting Qualitative Research (COREQ) (16). The study protocol was approved by the Ethical Committee of Shanghai Sixth People’s Hospital [No. 2022-KY-042(K)].

### 2.1. Sample and recruitment

Patient volunteers were those who assisted the medical staff in the FSH. Their duties included distributing supplies, maintaining order, cleaning and sterilizing, assisting with nucleic acid sampling, admission and discharge procedures, communicating information, etc. The interviews were conducted in West Bank FSH from May to June 2022. The West Bank FSH contained 1,356 beds admitting a total of 1,351 patients during the Shanghai pandemic. In a more than 1,000-bed FSH, there are typically about 20 patient volunteers in the Shanghai Pandemic. In the present study site, 21 patient volunteers were serving 1,351 patients. Purposive sampling was used to ensure the recruitment of patients of diverse age, education, and gender. Inclusion criteria were COVID patients, who (1) served as volunteers in FSH; and (2) were aged 18–60 years old. Exclusion criteria were COVID patients who (1) withdrew midway through the study; (2) had a history of mental illness; and (3) had communication disorders. When a patient volunteer is willing to be interviewed, a written document that describes the purpose of the study, the interview format, general interview questions, and our contact information will be provided. As the interviews were conducted online, oral consent and responses to multiple-choice informed consent questions in electronic questionnaires from patient volunteers were recorded by the interviewers. The participants had the right to decline participation or withdraw from the study at any time without any negative consequences.

### 2.2. Data collection

Semi-structured interviews were adapted to ensure flexibility. The interview guide was developed based on a literature review and discussions of the research group (See Supplementary material).

The video interviews were conducted at Tencent meetings when the volunteers finished their work or were discharged from the FSH. Their personal information was collected by the online questionnaire tool named “Questionnaire Star Application.” Interviews were carried out by three authors (ZLY, XMQ, and RTT). ZLY was a nursing management specialist with experience in conducting in-depth interviews and qualitative analyses on sensitive topics. XMQ and RTT were postgraduate students majoring in nursing, who had undertaken training and had experience in qualitative research. All the interviewers did not establish relationships with the participants prior to the interviews. To minimize the interruptions, all interviews were taken place in a quiet environment with only the interviewers and interviewees in the online meeting room. The interviews lasted between 25 and 60 min. There was a return to participants for comments and/or correction of transcriptions after each interview. Each interview was recorded and then transcribed manually sentence-by-sentence within 24 h after being finished. Data saturation was reached until no new information emerged in two consecutive interviews. When the 13th participant finished her interview, no new information emerged, and thus, the final sample size was 13. Two moderators (CJ and ZLY) iteratively assessed the content of case investigator sessions until no new themes emerged and separately followed the same process for contact notifier sessions. We did not conduct follow-up interviews or discussions and did not have participants review the transcripts.

### 2.3. Data analysis

Thematic analysis was applied to data analysis. We followed the six steps described by Braun and Clarke (17). We used MAXQDA 2020 for data analysis. At first, the first researcher imported the data into the software. Two researchers (XMQ and RTT) read the transcripts repeatedly to get the initial idea. Once they were familiar with the transcripts, they did the open coding in MAXQDA 2020. The codes were grouped to generate potential subthemes together. They checked subthemes with code extracts and generated a theme map, which was used to refine the themes by figuring out the relationships between different subthemes and the way they were formed by codes through mind maps. In this step, themes were also generated comprising relevant subthemes expressing similar central ideas. After reviewing the themes, they defined each theme and illustrated them with selected quotes. Finally, the coding results were reviewed and revised by another author (ZLY). All data analysis was completed in Chinese. Two authors (XMQ and RTT) fluent in both Chinese and English translated the codes and quotes into English. Another bilingual author (ZLY) checked the accuracy of the translation. Disagreements were solved by group discussion of the three authors until a consensus was reached. MAXQDA 2020 was used for data analysis. For data extraction and analysis, two methods were adopted to enhance validity: tier triangulation of the researchers and a well-documented audit trail of materials and processes.

## 3. Results

In total, 13 patient volunteers were included in this study, aged 18–53 years. Ten volunteers were male and three were female. Seven of them obtained junior college degrees or above. Four of them were workers, four were clerks, three were administrative staff, and two did freelance work. The length of stay in FSH ranged from 5 to 21 days. The volunteer time ranged from 4 to 20 days (Table 1). Four themes with nine subthemes were identified during the data analysis of this study (Table 2).

**Table 1:** Personal information of participants.

No.	Gender	Age (years)	Education level	Marriage	Occupation	Length of stay (days)	Volunteer time (days)
1	Male	32	Master	Married	Clerk	6	6
2	Male	19	Junior college	Unmarried	Freelance work	21	8
3	Female	47	Undergraduate	Married	Administrative staff	5	4
4	Female	24	Undergraduate	Unmarried	Clerk	15	13
5	Male	32	Undergraduate	Married	Clerk	18	18
6	Female	38	Master	Married	Administrative staff	16	14
7	Male	22	Junior high school	Married	Decoration worker	20	15
8	Male	35	Senior high school	Divorced	Administrative staff	16	6
9	Male	41	Primary school	Married	Construction worker	16	14
10	Male	53	Senior high school	Married	Freelance worker	20	20
11	Male	33	Junior high school	Divorced	Decoration worker	13	13
12	Male	28	Undergraduate	Unmarried	Clerk	10	8
13	Male	43	Junior college	Married	Cleaner	11	9

**Table 2:** Themes and subthemes.

Themes	Subthemes
Theme 1: Following the guidance of the heart	Subtheme 1: Giving back to the society
	Subtheme 2: The sense of political and religious responsibility
Theme 2: An irreplaceable role for Fangcang Shelter Hospital management	Subtheme 1: Distributing supplies
	Subtheme 2: Communication bridge between healthcare professionals and patients
	Subtheme 3: Providing psychological counseling for patients
Theme 3: Treating the work unruffled	Subtheme 1: Facing barriers in voluntary work
	Subtheme 2: Nothing special about voluntary work
Theme 4: Gifts from voluntary work	Subtheme 1: Benefits for physical and psychological well-being
	Subtheme 2: Another chance of growth

### 3.1. Theme 1: following the guidance of the heart

#### 3.1.1. Subtheme 1: giving back to the society

Most participants believed they should give it back to society, as they were admitted to the FSH with free food and accommodation. This was their main reason to become volunteers, though they were infected by COVID-19. “*I accompanied my wife to see a doctor in Shanghai, but we were infected with COVID-19 when staying in the hotel before seeing the doctor*. *When staying in the hotel, the neighborhood gave us food, as well as a lot of help*. *Fortunately, my wife and I were sent to the same Fangcang Shelter Hospital without separation*. *So, I joined the voluntary work on the first day of arriving at Fangcang Shelter Hospital*. *I wanted to give something back to the people around me (06)*.” Eight participants thought they were young and strong enough to serve the elders and children. “*Besides, there were a lot of elders and children in the Fangcang Shelter hospital*. *I thought I could help them (11)*.” Impressed by the hard work of the medical workers, 10 participants joined the voluntary team to relieve the burden of medical workers. “*After arriving at the Fangcang Shelter Hospital, I found that there were too many COVID patients and mass shortages of nurses*. *As there was a volunteer recruitment, I thought I should do it (13)*.”

#### 3.1.2. Subtheme 2: the sense of political and religious responsibility

The responsibility of the Chinese Communist Party members and the national responsibility forced the participants to participate in voluntary work. “*I am a party member and a veteran*. *On the first day of entering the Fangcang Shelter Hospital, I found they were recruiting volunteers*. *I was the first person to raise my hand to sign up, and I was thinking about reducing the burden on the medical staff*. *Later, when I saw that there were not many party members among the volunteers, I felt that I should stand up and take some responsibility (07)*.” For some participants, the religious doctrine required them to devote their love to others. Through voluntary work, they got peace of mind through performing the religious doctrine. “*Christians need to give some love in behavior*. *Since there was a need for volunteers in Fangcang Shelter Hospital and we were young people, we needed to pay more*. *I feel very happy about this work*. *In our words, it is to glorify God! (13)*.”

### 3.2. Theme 2: an irreplaceable role for Fangcang Shelter Hospital management

#### 3.2.1. Subtheme 1: distributing supplies

Supply distribution was the major part of their work. Volunteers were responsible for the distribution of meals, fruits, drinks, medicine, and other daily necessities. “*Our first task is to distribute food for everyone, in order to avoid the occurrence of crowding and reduce the risk of cross-infection (02)*.” Some volunteers brought the experience of human resources management into their voluntary work to improve the work efficacy. “*I write down the food-sharing experience and print them out, and then tell everyone, especially the new volunteers*. *Anyway, I think the collective wisdom can be preserved, although meal distribution is only a small thing (12)*.”

#### 3.2.2. Subtheme 2: communication bridge between healthcare professionals and patients

Volunteers served as the connection between healthcare professionals and other COVID patients. They communicated with the patients more easily than the medical workers. They could send messages about the needs and difficulties of COVID-19 patients to the medical workers and hospital managers, which saved communication time and reduced conflicts during communications. “*Older people are not mobile*. *We (volunteers) need to take him to the bathroom and deliver their food to their beds*. *They would call us if they had any discomfort*. *Then, we will contact the doctors and nurses (12)*.” They could also give the notice of medical workers to the patients, making it easier to understand and accept. The volunteers found that they were more capable than the doctors and nurses in communicating with other COVID-19 patients. “*I think my biggest contribution is to communicate with the patients and make them understand the requirements of doctors and nurses*. *Since we are all patients, it is better for me to talk to them*. *For example, at the beginning, some people did not understand the requirements of the PCR test, but I used the identity of the patient to talk with them and they understood the requirements more smoothly*. *I feel that I can share the work with the medical staff*. *It is very meaningful (07)*.”

#### 3.2.3. Subtheme 3: providing psychological support for patients

Doctors were responsible for identifying mental health problems and referring them to specialist clinics. Some patient volunteers provided temporary psychological support in the FSH as there were few professional therapists. As these volunteers were also patients, they used their own experiences to encourage the patients in the FSH. They helped other COVID-19 patients to reduce anxiety and tension and to adapt to the environment of the FSH. Their common goal was to be discharged soon. There was no potential harm to the patients when these patient volunteers provided psychological support. The FSH management organized weekly online meetings with the patient volunteers in order to be aware of their needs and to help them deal with their problems. These patient volunteers worked with the nurses and logisticians. The head of the nursing team and the head of the logistics team also worked with these volunteers, giving them guidance at all times. The research was also reviewed by the hospital’s internal ethics committee before the formal study was carried out. “*Most patients coming to the Fangcang Shelter Hospital were depressed*. *I went to talk to the patients who were not far from my bed: ‘COVID-19 is not so horrible’ (03)*” The volunteers were mostly in relatively good mental state, and hoped to influence other COVID patients to make them optimistic about the diseases and life in FSH. “*I am quite optimistic*. *I also hope to pass the positive emotions and energy to influence others around me (05)*.”

### 3.3. Theme 3: treating the work unruffled

#### 3.3.1. Subtheme 1: facing barriers in voluntary work

Like most of the other work, various challenges were faced by the patient volunteers during their voluntary work and they tried to overcome the difficulties to accomplish the work. The instability of the volunteer team was one of the biggest difficulties they faced. The short recovery period for COVID-19 infection led to the relatively short stay in FSH of the patient volunteers. In most cases, it took several days for the volunteers to be trained to get familiar with their work, while they were about to be discharged at that time. Since the volunteers will be discharged when they are healed, the team is unstable. Nevertheless, we were able to recruit new patient volunteers and organize handover activities by introducing recruitment activities during admission orientation when admitted to the FSH and by posting detailed messages on the communication wall in the FSH. “*Perhaps, the only problem we encountered is that the infection of coronavirus is a cycle, usually lasting for 10 days to 2 weeks*. *Sometimes, the volunteers are still here today, and then they will get discharged tomorrow*. *There is a fast turnover rate for the volunteers (02)*” Some volunteers found it hard to find new volunteers to take over their job when they were going to be discharged. The lack of willingness to participate in the voluntary work of other COVID-19 patients troubled the volunteers. “*At first, people did not understand the voluntary work*. *Very few people took part in it, and no one understood it*. *In the beginning, people did not really understand what I said, because I was not only a volunteer, but also a COVID patient as well (03)*.” The age, occupations, and education level varied among volunteers, who held different world views and worked in different ways. As strangers working together, volunteers faced difficulties in coordinating with each other. Sometimes, conflicts about working ways existed in the team of volunteers. “*Sometimes, there were some problems in our cooperation*. *I had distributed food in this row of beds, but he (another volunteer) did not know what I had done and did it again*. *People were often confused with the work (05)*.”

Health problem was another concern of volunteers. Some of them worried about their recovery time after taking over the voluntary work, as they had to get in contact with other COVID patients. “*So many COVID patients were together in Fangcang Shelter Hospital*. *The concentration of the virus was quite high, and the environment was very different (from the outside world) (07)*.” Besides, several volunteers complained that their COVID symptoms troubled them and forbade them from doing the voluntary work, but they overcame the physical discomfort and completed their work. “*But I actually have some symptoms*. *I cough a lot, and really could not talk, so I did some work without the need to talk with others*. *The early days of my voluntary work were like this (09)*.”

#### 3.3.2. Subtheme 2: nothing special about voluntary work

Participants viewed the voluntary work in FSH as ordinary work. They thought their work was easy, and spent limited physical or mental energy. “*There were no huge obstacles in voluntary work*. *It was just a normal work (01)*.” Some participants reported they did not struggle in the decision to participate in the voluntary work. “*I was transferred to the Fangcan Shelter Hospital on the 21st*. *On the same day, I saw a doctor who needed help with PCR tests, so I went straight to help him*. *In fact, there was nothing, no big changes in my mind (01)*.” Despite the panic they had when first arriving in the FSH, some volunteers changed their views about COVID and became relaxed after being familiar with the environment. The shift in mindset made them join in the voluntary work, as they viewed the FSH as a normal social environment. “*On the first night living in Fangcang Shelter Hospital, I could not sleep*. *The next day, I slowly calmed down*. *I knew that so many people were admitted to Fangcang and many people were discharged*. *The disease was not so terrible*. *I talked to myself ‘I’m staying here anyway, so I should do something meaningful’*. *Then I applied to be a volunteer and continued to work! (03)*.”

### 3.4. Theme 4: gifts from voluntary work

#### 3.4.1. Subtheme 1: benefits for physical and psychological well-being

Voluntary work provided participants with opportunities to get in touch with others in a new environment, including COVID patients and medical workers. They were also involved in the management affairs of FSH. Serving as volunteers in FSH retained the social function of the patient volunteers, helping them go back to social life, instead of being treated like sick patients. Voluntary work filled their quarantine life, making the quarantine life more interesting and meaningful. “*The days as a volunteer are more fulfilling than before, giving me more pleasure (04)*.” Voluntary work made them relaxed and relieved their psychological burden. One participant stated doing voluntary work gave them peace of mind. “*When I began to do something meaningful, like helping others do something in Fangcang Shelter Hospital, I began to feel nothing horrible*. *Gradually, my mind became peaceful (03)*.” Some participants believed that being volunteers made them exercise more and stay positive, which helped them with rehabilitation from COVID. “*Maybe I can encourage myself by volunteering*. *I feel happy for myself as I can help others*. *Once you are in a good mood, you’ll probably recover a little faster*. *Some people here, you know, just lay on the bed playing with their cell phones*. *In fact, I do not think it is beneficial to his recovery (05)*.”

#### 3.4.2. Subtheme 2: another chance for growth

Voluntary work in FSH increased the life experience of volunteers, bringing them another chance for growth. “*At first, voluntary work changed my mind and style*. *I used to muddle along from day to day, but at least now there is something meaningful that I think I can do in the future, like charity*. *I will continue to do it (04)*.” Their interpersonal communication experience was gained through working with all kinds of people. Volunteers viewed their work as a special and meaningful experience for themselves, which was also educational for their children. “*My son had never seen me giving something to others, but he probably knew I was doing the voluntary work*. *He did not know exactly what I was doing and just had such an impression*. *I want to set an example for him like this (12)*.”

Working as volunteers in FSH broadened the horizons of the patient volunteers, letting them know more about the hardships of the healthcare providers and be more considerate of them. They understood the difficulties in epidemic prevention and management and were willing to protect the medical staff and devote themselves to voluntary work. “*It was raining heavily that day, and the medical staff was still working as usual*. *I was very impressed and touched*. *Also, it was very hot in the cabin*. *Everyone was dressed in protective clothing, and they were soaked from the inside out*. *However, they did not complain*. *They still did what they had to do*. *This job was really hard*. *It was not easy for you (07)*.” Volunteers paid more attention to other patients and learned to take care of them. “*Right*. *There is an office for staff*. *Every time I go to the bathroom or whatever*. *I will look over the office to find out if anybody is looking for help (09)*.” The sense of responsibility brought by the nature of their voluntary work increased among the volunteers. The volunteers were impressed by the unity of the staff team in FSH, viewing the medical workers and patient volunteers as a whole family. “*Medical staff, volunteers, and patients, we all became friends*. *We were roommates and colleagues in Fangcang Shelter Hospital and friends when we went out (04)*.”

Volunteers also received recognition and support from multiple parties, including other patients and health professionals. “*Firstly, I get the approval of others*. *This kind of recognition is not the approval of the boss at work*. *It is the approval from people who do not know each other, which is another kind of recognition*. *I do not know how to express this feeling*. *It’s just a little sense of achievement, but it is not vanity (09)*.” The cooperation from other volunteers created a harmonious team working environment, improving the efficiency of voluntary work. “*I also gained comrades in arms in Fangcang*. *We are all working for a common goal and I hope that the spirits of volunteers can be passed on to more people (02)*.” The joy of helping others increased the sense of self-identity of patient volunteers, which motivated them to devote more to their work.

## 4. Discussion

In this study, we explored the experience of patient volunteers in an FSH during the Omicron wave of the COVID-19 pandemic in Shanghai. As an emerging and unique group in China’s fight against the COVID-19 pandemic, we highlight the key roles of the patient volunteers and the responsibilities they fulfilled during this period. Furthermore, we outline the important considerations for the ethical use and safety of patient volunteers. To the best of our knowledge, this is the first study to provide novel insight into the patient volunteers’ work and life in FSH. Besides, we identified some barriers when carrying out volunteer activities during the public health emergency, including some facilitators and potential solutions to improve voluntary work engagement.

In this study, we found the desire to give back to society, as well as political and religious responsibility, were the major reasons for becoming patient volunteers in the FSH. The responsibility of the Chinese Communist Party members and religious beliefs encouraged the participants to join the voluntary work. Four sources of motivation are identified from the literature, including individual motivation, family motivation, community motivation, and organizational motivation (18). Accounted by Clary and Snyder, the volunteering motivation that emerged in various crisis situations was divided into six types, including values, understanding, enhancement, career, social, and protective (19). Under the context of Chinese culture, altruism, and collectivism are viewed as the dominant motivation, which advocates selfless dedication to others and society (20). Concerning the motives of the patient volunteers in FSHs, altruism motivated them to cope with the acute crisis imposed on public welfare services, rather than monetary remuneration reported as the main motivation in other studies (21, 22). Furthermore, research found that a significant positive relationship between the medical students’ religiosity and their eagerness to commit for the sake of the community during COVID-19 rather than for personal or egoistic motives (23). Although patient volunteers’ religiosity was not a significant predictor of volunteering during the COVID-19 outbreak, it influenced their motivations to join the fight against the pandemic. It seems that particularly during the current health crisis caused by the COVID-19 outbreak, religion strengthened ordinary people’s perception of the medical profession as a unique vocation and moral service. They felt it was their duty to engage with and help those in need, disregarding the risk. Nevertheless, even in the case of patients who defined themselves as deeply religious, pure altruism was not the only motivation, as most respondents volunteered to gain skills, connections, or some kind of psychological satisfaction. Thus, it should be acknowledged that the motivations of many patient volunteers were often a mixture of altruistic and egoistical drivers. However, for many volunteers, religion served as a unique protective factor to overcome stress, making them feel more competent to become volunteers. Altruism was more likely to exist in the patient volunteers with religious beliefs. Additionally, personal health issue was mostly a concern for people to become volunteers. The current study showed that participants who perceived themselves at risk of contracting COVID-19 were less likely to become volunteers. In line with this finding, most volunteers perceived themselves as strong and healthy in our study (24).

Finding successors when discharged from the hospital was identified as one of the main difficulties for the patient volunteers in maintaining the continuity of voluntary work. During the Omicron wave in Shanghai, patient volunteers in FSHs were mostly viewed as temporary jobs without formal training and certifications, which may lead to unstable volunteer groups. Our findings highlighted the need to establish a volunteer management team in FSHs to ensure their collaboration, coordination, and regularly updated communication with the hospital managers. The team should serve as a bridge between the patients and hospital managers, involved in the details of operational processes, and facilitating smoother volunteer recruitment, training, placement, and coordination. During the volunteer recruitment, the organizational commitment of the volunteer programs and the support from the medical staff should be announced by the volunteer management group (25). Long et al. (26) emphasized that the first action of such a team was to ensure regulatory and safety measures were in line with the legal parameters and patients’ capacity. The recruitment of volunteers is a common problem in volunteer management programs. In addition to understanding the motivations of volunteers and providing continuous training focusing on the volunteers’ roles and the skills they need, arranging the volunteer agenda according to the schedules of volunteers is also found as the facilitators in recruiting and retaining the volunteer groups (25).

The patient volunteers played a wealth of alternative roles as they can contribute to the management of FSH and the support of the quarantine of COVID-19 patients. Volunteers are crucial in the prevention and control of the COVID-19 pandemic. Among them, the majority are healthcare professionals and medical students. They have undergone professional training, and are able to undertake various medical professional work, such as hospital work, call center and administration, epidemiological aspects, and laboratory-related work (27, 28). However, the shortage of healthcare professionals around the world makes it difficult to accomplish the volunteer tasks, while the patient volunteers made up for the shortage of volunteers. Though the patient volunteers are not healthcare professionals, they helped alleviate the workload of the medical staff in FSH. Notably, they played crucial roles in the physical comforting of other COVID patients in FSH, even more advantageous than the medical staff. Providing psychological care as a part of peer support, patient volunteers benefited the psychological health of other patients and accelerated their recovery of themselves (29). The mutual help between patients has been proven effective in addictive disorders, mental health problems, and cancer patients (30, 31).

The interests and goals of volunteers and patients are aligned. When working with medical staff, the patient volunteers were protected the same as ordinary patients. They acquired adequate training prior to the beginning of the job, *in situ* guidance and problem-solving, psychological support. Volunteer certificates were issued upon completion of the work to show appreciation and respect on behalf of the team.

The patient volunteers in this study discussed the impacts of voluntary work on psychological well-being, illness attitudes, and behaviors. The development of personal and interpersonal skills such as adaptability, leadership, and ethical commitment has been promoted during the voluntary work. Though COVID-19 left the patients with physical pain, mental burden, and inconvenience in daily life, some patient volunteers believed the voluntary work helped reduce negative emotions such as anxiety and depression, and can also increase the sense of self-worth. The benefits of voluntary work have been proven in existing studies, including increasing the sense of pleasure and lowering the risk of mortality (32). The sense of belonging to the profession of the patient volunteers was cemented as they accumulated social experiences through volunteering, and received consistent support and recognition from healthcare staff and the general public. Our findings were consistent with the previous studies that warm welcome by healthcare staff, amicable staff interactions, and behaviors, team comradeship, and appreciation are components instrumental in positive volunteer relationships cultivating a sense of belonging and sustaining the motivations of volunteering (33). Participating in the voluntary work was also an opportunity for the COVID patients to get familiar with the new environment and medical workers, change their stereotypes about healthcare work, and relieve their worry about the quarantine life in the FSH. Compared with people isolated at home, patient volunteers fighting against the COVID-19 pandemic gave them a sense of purpose and social responsibility, which made them more positive about the quarantine life in the FSHs. This condition may increase their interest in volunteering throughout this period. In addition, previous studies suggest that social interactions play an important role in the willingness to be a volunteer, which is more significant than the activity itself (34). Therefore, it is crucial to sustaining the commitment of patient volunteers by promoting a cohesive and positive relationship between patient volunteers, facilitating clinical guidance by allocating preceptors and providing psychosocial support and close follow-up with patient volunteers’ interactions with medical staff.

Though this study provided a novel view of the patient volunteers in FSH, limitations still existed. This study only included patients from one FSH, while the experience of patient volunteers from other FSHs should also be explored as the differences in management of different FSHs. As the results were mainly obtained from the self-reported information, recall bias might exist.

## 5. Conclusion

This study provides unique insights into the experiences of FSH patient volunteers during the COVID-19 pandemic in Shanghai. At the individual level, patient volunteers demonstrated their “volunteer” identity through acts of assertiveness, compassion, competence, confidence, conscience, commitment, and courage during the pandemic. On an interpersonal level, they experienced continuous personal growth, maturity, and validation through professional socialization as their sense of belonging was strengthened. At the societal level, COVID-19 provided an opportunity for volunteers to develop specialized skills in infectious disease preparation. The skills of emergency management are useful for the volunteers. They supported the medical staff at FSHs and effectively reduced the volatile social crisis. Recruiting patient volunteers with mild diseases could address the shortage of medical personnel and reduce the burden on the healthcare system during public health emergencies.

Future studies can follow the psychological course and effects on patient volunteers over time, and explore the changes in different periods to deepen the understanding of volunteerism in public health emergencies. In addition, subsequent researchers can propose a reasonable training model for patient volunteers to improve their ability to cope with public health emergencies.

## 6. Limitations

Although the sample is as diverse as possible, there may still be selection bias. Recall bias may exist when patient volunteers were asked to recall the details of their work and experiences. Due to time constraints, we only included patient volunteers from one FSH, which may not be representative enough.

## Data availability statement

The original contributions presented in the study are included in the article/**Supplementary material**, further inquiries can be directed to the corresponding author.

## Ethics statement

The studies involving human participants were reviewed and approved by the Ethical Committee of Shanghai Sixth People’s Hospital [No. 2022-KY-042(K)]. The patients/participants provided their written informed consent to participate in this study.

## Author contributions

LZ and JC: conception. LZ, JC, and MX: methodology. LZ, MX, TR, and XH: data collection. LZ, MX, and TR: data analysis. LZ and MX: manuscript drafting and reviewing. All authors contributed to the article and approved the submitted version.
