# Supplementary material for: The role of patient volunteers in Fangcang Shelter Hospital during the Omicron wave of COVID-19 pandemic in Shanghai, China: a qualitative study
Source: Front Public Health. 2023 Oct 12;11:1215030. doi: 10.3389/fpubh.2023.1215030 (PMC10600484; doi:10.3389/fpubh.2023.1215030)
Supplement: Supplementary file 1 [file Data_Sheet_1.DOCX]

**Interview guide**

**Main questions**

1. Would you please tell us the reason and the process of becoming a volunteer in the Fangcang Shelter Hospital?

2. Could you talk about your transition in mind from a patient to a volunteer?

3. What are your main responsibilities in volunteer service in the Fangcang Shelter Hospital?

4. What is your experience of volunteering in the Fangcang Shelter Hospital? How has the state of mind and mood changed?

5. What have you learned from the voluntary work in the Fangcang Shelter Hospital?

6. What problems do you think exist in the management of volunteers in the Fangcang Shelter Hospital? Please make some suggestions.

**Follow-up questions**

7. What are your concerns about voluntary work in the Fangcang Shelter Hospital?

8. What do you think would motivate you to become a volunteer in the Fangcang Shelter Hospital?

9. What difficulties do you encounter in voluntary work in the Fangcang Shelter Hospital? How did you handle them?

10. What kind of support do you get from others during the voluntary work in the Fangcang Shelter Hospital?

11. Please share with us your unforgettable experience of the voluntary work in the Fangcang Shelter Hospital.

12. Will you continue to work as a volunteer for the COVID-19 prevention and control? Please explain the reasons.
